# Construction and Performance of Superhydrophobic Surfaces for Rusted Iron Artifacts

**DOI:** 10.3390/ma16062180

**Published:** 2023-03-08

**Authors:** Pei Hu, Minghao Jia, Hao Xu, Xiaogu Zhang, Dongbo Hu, Gang Hu

**Affiliations:** 1 School of Archaeology and Museology, Peking University, Beijing 100871, China; 2 The Palace Museum, Beijing 100009, China

**Keywords:** ancient iron artifacts with rust, tannic acid, stearic acid, modified superhydrophobic

## Abstract

Ancient iron artifacts need to be protected with a rust layer, often stabilized by tannic acid corrosion inhibition. In humid environments, water vapor could slowly penetrate and trigger galvanic corrosion of metal artefacts. Sealing treatments are generally applied to the artefact surface to isolate water and enhance its corrosion resistance. Superhydrophobic modifications could effectively block the penetration of moisture into the interior of the artefact and provide a nice water barrier. Stearic acid with tannic acid inhibition treatment creates a superhydrophobic protective layer on the surface of rusted iron artifacts and enhances corrosion resistance effectively. Various scientific analyses and testing methods are used in this paper to evaluate the corrosion resistance of rusted surfaces after superhydrophobic modification and investigate the reaction mechanisms. The results indicate that the contact angle of the rusted surface after corrosion inhibition by tannic acid and modified by stearic acid is increased to 152.2°, which means the superhydrophobic protective layer has been successfully constructed. The C/Fe ratio of the rusted surface is increased from 0.21 to 2.10, and the characteristic diffraction peaks of O1s and Fe 2p_3/2_ shift toward higher binding energy. Stearic acid is combined with the corrosion product layer by chemical bonding. Chelation between rust products, tannic acid, and steric acid is effective, and the chelate is chemically stable. The superhydrophobic surface forms a lamellar wax-like layer as an air barrier to isolate liquid water, resulting in a significant decrease in corrosion current and an increase in Warburg impedance to 217.9 times the original state, with a protection efficiency of 88.3%. Tannic acid corrosion inhibition and stearic acid superhydrophobic modification have an excellent synergistic protective effect on improving the corrosion resistance of iron artifacts, resulting in better corrosion resistance of iron artifact materials. The research provides new ideas and references for the protection of ancient iron artifacts sealing.

## 1. Introduction

Iron relics are essentially historical and cultural carriers of ancient civilizations with precious historical, artistic, and scientific value. However, the chemical properties of iron are pretty active, and iron heritage artifacts are subject to rapid oxidation in the preservation environment, resulting in severe corrosion damage. In general, iron artifacts need to be protected with a rust layer, while corrosion products are hydrophilic. With the increase of the atmosphere’s relative humidity, the water film thickness on the surface of iron artifacts also increases. As atmosphere pollutant particles and harmful gases dissolve in the water film, forming an electrolyte solution, the transformation of iron artifacts from chemical corrosion to electrochemical corrosion, resulting in a corrosion rate, increased significantly [1]. Therefore, water is one of the most critical factors affecting the stable preservation of iron artifacts. It is suitable for constructing a superhydrophobic protective layer on the surface of ancient iron artifacts for sealing treatment.

Superhydrophobicity is a special infiltration phenomenon on the surface of many living organisms in nature and has been one of the hottest research topics in materials science in recent years. Superhydrophobic surfaces are those with a water contact angle greater than 150° and a sliding angle less than 10°. The coating provides an extraordinary superhydrophobic property, effectively preventing the adhesion and penetration of liquid water and condensation of water vapor. The corrosion resistance of the metal matrix is improved by reducing the contact area between the corrosive medium and the metal matrix, preventing the penetration of the corrosive medium [2,3]. Extensive studies in the fields of anti-fouling and self-cleaning [4,5], water and moisture resistance [6,7], anti-icing [8,9], and coating protection [10] have shown the significant advantages of superhydrophobic surfaces in preventing the atmospheric corrosion of metals [11,12].

Superhydrophobic surfaces generally have two main characteristics, hierarchical micro/nanostructure and low surface energy [13,14], which determine the good or bad material wettability [15]. The preparation processes include etching [16], immersion [17], deposition [18], sol-gel [19], spraying [20], and so on. Among them, the immersion method can be conducted with simple reaction conditions and equipment; the sol-gel method does not require physical or chemical treatment of the substrate; the spray method is more suitable for the surface of artifacts since it does not depend on the substrate [21,22]. However, the construction of iron-based superhydrophobic surfaces generally aims at bare iron surfaces, such as electrodeposition [23,24,25] and spraying methods [26,27,28], to construct superhydrophobic protective layers on mild steel surfaces. In contrast, the surface of iron artifacts is usually covered with a corrosive layer consisting of iron oxides and hydroxides, which need to be protected with rust.

In combination with the current primary conservation process of iron artifacts, rusted iron artifacts are often treated with corrosion inhibiting materials such as tannic acid and then surface sealing [29]. For the protection of ferrous materials, tannic acid is commonly used for corrosion inhibition [30,31], and stearic acid for low-surface energy modification [32,33], showing good corrosion resistance. Tannic acid can chelate with iron ions to generate iron tannate covering the surface and transfer active corrosion products into a stable state. Stearic acid can graft to the surface to improve hydrophobicity. The protective layer’s composition and morphology will change after treatment, while the metallographic structure will not. It complies with the requirements of the conservation of ancient metal artifacts. This study hopes to construct a superhydrophobic protective layer on the surface of rusted iron artifacts to improve anti-wetting properties, reduce the contact area between liquid water and the metal matrix, slow down or prevent the formation of electrolyte water film on the surface, and improve the corrosion resistance of rusted iron artifact materials. The life of iron artifacts will be extended by improving the corrosion resistance of materials with rust.

## 2. Materials and Methods

### 2.1. Specimen Preparation

Preparation of the rusted surface: The iron samples were polished by SiC paper (grade 180–1000), ultrasonically cleaned with anhydrous ethanol and deionized water then dried. To prepare a uniform thin rust layer covering the surface, the samples were brushed with 15% FeCl_3_ after acid etched in 5% H_2_SO_4_.

Preparation of the superhydrophobic surface: the rusted iron samples and ancient iron coins were immersed in 3% tannic acid aqueous solution for 1 h at a room temperature of 25 °C, then water bathed in 10% H_2_O_2_ at 50 °C for 10 min. After ultrasonic cleaning, the samples were placed in an oven at 120 °C for 4 h to remove water, immersed in stearic acid alcohol solution for 15 min for modification, and finally cured at 100 °C for 1 h to produce a superhydrophobic surface [34,35,36].

### 2.2. Characterization

The analytical instruments used for the study are listed below:

Contact angle meter: The contact angles (CAs) of rusted iron samples before and after protection treatment were measured by a contact angle meter (DSA30, KRUSS, Hamburg, Germany) at room temperature with a droplet volume of 3 μL. Three measurements on different areas of the surface were taken and the average value was taken as the test value.

Scanning electron microscopy (Quattro ESEM, Thermo Fisher, Waltham, MA, USA) was used to observe the surface morphology of rusted iron samples before and after protection treatment under a scanning voltage of 15 kV and in high vacuum conditions.

FTIR transmittance analysis (Tensor 27, Bruker, Karlsruhe, Germany) was used to test the structural information of the rust powder before and after protection. A rust powder sample of 0.2 mg mixed with 200 mg of dry KBr (>99% FTIR grade, Sigma-Aldrich, Burlington, MA, USA) were milled and pressed into pellets and tested under FTIR in the range from 400 to 4000 cm^−1^ at 4 cm^−1^ resolution.

X-ray photoelectron spectrometer (XPS): XPS (AXIS Supra, Kratos Analytical Ltd., Manchester, UK) was used to analyze the elemental composition and chemical state of the surface. Aluminium was used as an X-ray photon source with the power of 150 W. The pass energy in the full spectrum was set at 160 eV with an energy step of 1.0 eV. The pass energy in C, O, Fe elemental spectra was set at 40 eV with an energy step of 0.1 eV.

### 2.3. Evaluation of Corrosion Resistance of Rusted Iron Samples

In order to evaluate the corrosion resistance of rusted iron samples after superhydrophobic modification, the rusted iron samples are examined by polarization curves and electrochemical impedance spectroscopy.

Electrochemical test system: The polarization curves and electrochemical impedance spectra of the rusted iron before and after protection were examined by an electrochemical workstation (CS-350, CorrTestTM, Wuhan, China). A three-electrode system was used: a rusted iron sample as the working electrode (with a working area of 1 cm^2^), a saturated calomel electrode filled with saturated KCl solution as the reference electrode, and a platinum electrode as the auxiliary electrode. Measurements were carried out in 3.5 wt. % NaCl solution as an electrolyte at room temperature. To stabilize the corrosion potential, the electrodes were immersed in the solution for 40 min before testing. The potential scanning rate of potentiodynamic polarization was 0.5 mV/s in the range from −200 to +500 mV (relative to the open circuit potential). The test frequency of electrochemical impedance spectra ranged from 10^−2^ Hz to 10^5^ Hz with the sinusoidal perturbation signal of 10 mV amplitude. The data collected was interpreted by Cview and Zview (Cview2/Zview2, Scribner Associates Inc., Southern Pines, NC, USA) based on an equivalent circuit in order to obtain the fitting parameters.

The ancient iron coins were subjected to alternating wet-dry salt spraying with 3.5 wt. % NaCl solution for five cycles to accelerate corrosion. One cycle of spraying included two stages: the spraying stage with a relative humidity of 99% for 8 h and the drying stage with a relative humidity of 30% for 16 h.

## 3. Results and Discussion

### 3.1. Corrosion Inhibition and Superhydrophobic Modification of Iron Samples with Rust

**Figure 1:**
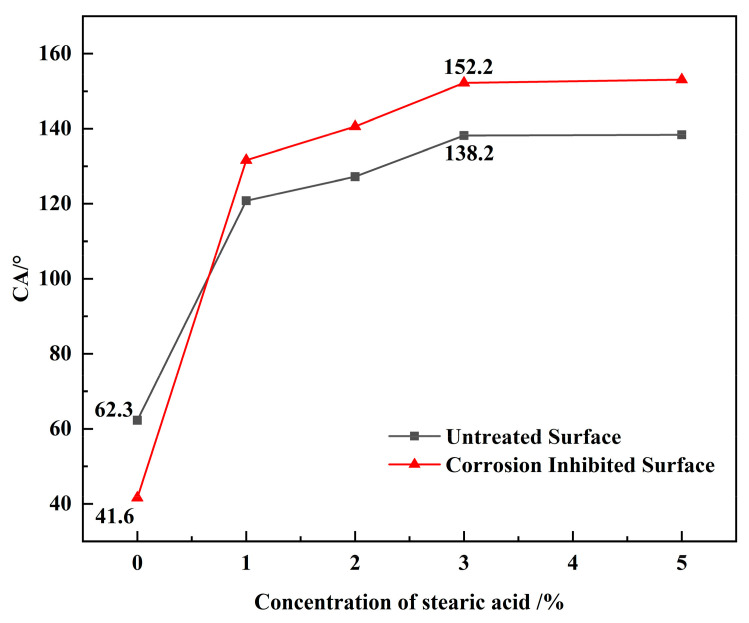
Contact angles of a rusted surface with different concentrations of stearic acid.

Figure 1 shows the water contact angle of rusted iron samples modified with different concentrations of stearic acid. As can be seen in Figure 1, the construction of superhydrophobic coating on the surface of iron samples with rust can be achieved by corrosion inhibition of tannic acid and modification of stearic acid. The water contact angle of the untreated rusted surface is 62.30°, and reduced to 41.6° after corrosion inhibition treatment of tannic acid. Hydrophilicity of rusted iron samples is increased slightly after tannic acid treatment, which might relate to a large number of hydroxyl groups of tannic acid.

The water contact angle of the rusted surface shows a large improvement after modification of the stearic acid, whether with corrosion inhibition treatment or not. For example, modified with 1% stearic acid, the water contact angle is increased to 120.8° for an untreated rusted surface, and increased directly to 131.6° for a rusted surface after tannic acid treatment. With an increase of concentration of stearic acid, the water contact angle further increases, and the hydrophobic properties of the rusted iron surface are all improved. With an untreated rusted surface, the water contact angle increases dramatically from 120.8° to 138.2° when the concentration of stearic acid is increased from 1% to 3%. When the concentration of stearic acid increases to 5%, the water contact angle increases to 138.4°, which does not change much compared to 3% stearic acid solution. That is because the rust layer contains many hydroxyl iron oxides, and the carboxyl groups of stearic acid can be bonded to the surface through a dehydration condensation reaction. The long alkyl chains provide good hydrophobicity. The rusted surface initially exposes a large number of hydroxyl reaction sites. When the concentration of stearic acid is lower than 3%, the higher the concentration, the more the alkyl chains that are grafted to the surface. The hydrophobicity has been improved, and the contact angle has increased greatly. When the stearic acid concentration reaches 3%, the surface has grafted many long alkyl chains, which means fewer hydroxyl reaction sites remain. Thus, the concentration continues to increase with little change in hydrophobicity. As for rusted surfaces after corrosion inhibition treatment of tannic acid, when the concentration of stearic acid increases from 1% to 3%, the water contact angle increases from 131.6° to 152.2°, which completes the construction of the iron-based superhydrophobic surface. Similarly, the water contact angle increases slowly by continuing to increase the concentration of stearic acid. When the concentration of stearic acid is higher than 3%, the growth of the water contact angle is very small, as is the concentration increase. The higher the concentration of stearic acid, the more difficult it is to dissolve, and it is considered that the more suitable concentration of stearic acid modification is 3%.

It can be seen that tannic acid corrosion inhibition and stearic acid modification are beneficial to enhance the superhydrophobicity of rusted surfaces, and the two have some synergistic effects.

### 3.2. Evaluation of Corrosion Resistance after Superhydrophobic Modification with Rust

#### 3.2.1. Polarization Curves

**Figure 2:**
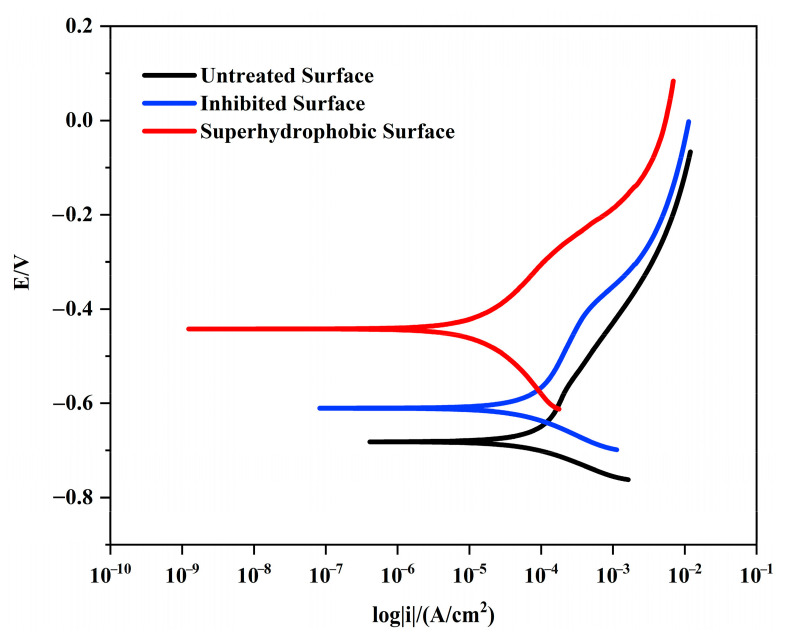
Potentiodynamic polarization plots of rusted iron samples before and after protection.

Figure 2 shows the polarization curves of rusted iron samples in different treatment states. The polarization curve shifts significantly in a positive direction after tannic acid treatment compared to the polarization curve of the untreated sample. After stearic acid modification, the polarization curve becomes increasingly positive, and the cathodic Tafel slope significantly increases. The polarization curve data and relevant electrochemical parameters are listed in Table 1. For untreated surfaces, the free-corrosion potential is −680.28 mV and the corrosion current is 111.51 μA·cm^−2^. For the corrosion inhibited surface, the free-corrosion potential is positively shifted to −609.59 mV. After corrosion inhibition by tannic acid and modification by stearic acid, the free-corrosion potential is positively shifted to −441.83 mV and the corrosion current is decreased to 13.009 μA·cm^−2^. The corrosion resistance is significantly increased, about 8.57 times that of untreated samples, resulting in a significant decrease in corrosion rate and a protection efficiency of 88.3%. It can be seen that the corrosion resistance of rusted iron samples has clearly improved after superhydrophobic modification. The polarization curve experiments were performed in 3.5% NaCl solution with iron samples covered in an iron tannate and superhydrophobic layer. The anodic and cathodic reaction processes are not changed, and are still Fe→Fe^2+^ + 2e^−^ and O_2_ + 2H_2_O + 4e^−^→4OH^−^. Due to the formation and coverage of the corrosion inhibition layer and the superhydrophobic layer, the exchange of ions in the electrode process has been prevented effectively, which shows an excellent effect in slowing down the corrosion. From the polarization curve, it can be seen that the corrosion potential is positively shifted, and the corrosion current is significantly reduced.

#### 3.2.2. The Electrochemical Impedance Spectroscopy (EIS)

**Figure 3:**
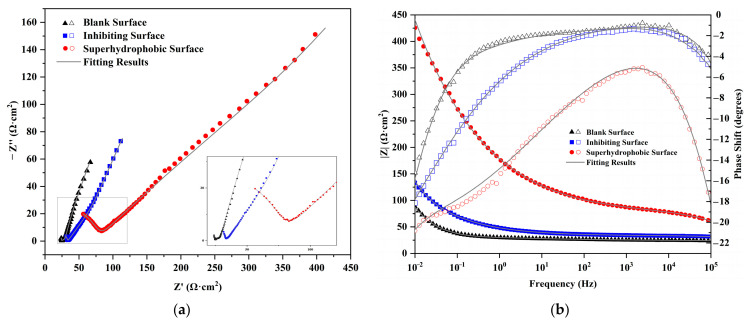
Nyquist (**a**) and Bode (**b**) plots of rusted iron before and after protection.

Figure 3 shows the electrochemical impedance spectroscopy of the rusted iron interface in the 3.5% NaCl solution under three different treatments. The EIS results of all three conditions exhibit the double capacitance semi-arc and a large Warburg impedance.

Figure 3a Nyquist plot and Figure 3b Bode plot show the existence of two time-constants for this corrosion system. The solution resistance and rust resistance dominate at the high frequency region from 10^4^ to 10^5^ Hz and form an incomplete capacitive semi-arc. The electrochemical processes and mass transfer diffusion processes on the surface of the iron dominate at the medium-low frequency region and form a large Warburg impedance curve. It is proved that the protective layer inhibits the free diffusion of electrolyte ions and internal metal ions.

**Figure 4:**
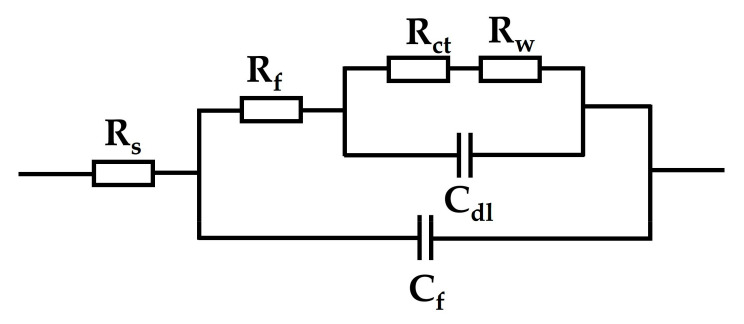
Equivalent circuits of EIS curves of rusted iron in a mixed solution.

The characteristics of impedance spectrum coincides with the corrosion state of rusted iron samples, and the impedance spectrum of corrosion-inhibited surfaces and superhydrophobic surfaces is similar to that of the untreated surface. It can be seen from Figure 3 that the shape of the impedance spectrum is not obviously changed before and after protection. This indicates that the combination of tannic acid and stearic acid with the rust layer does not completely change the reaction mechanism of corrosion, but only adsorbs on the rusted surface to form a protective thin layer to prevent the contact between the metal matrix and the corrosion solution. For that, the three impedance spectra are analyzed and the corresponding equivalent circuit is shown in Figure 4. In equivalent circuits, R_s_ represents the electrolyte resistance, R_ct_ the charge transfer resistance, C_dl_ the double-layer capacitance, R_f_ the rust resistance, C_f_ the rust capacitance, and R_w_ the Warburg resistance or barrier diffusion impedance. The electrochemical impedance spectroscopy is fitted based on this equivalent circuit, and the corresponding electrochemical parameters are listed in Table 2.

For superhydrophobic surfaces, R_ct_ increases from 3.537 Ω·cm^2^ to 82.38 Ω·cm^2^, an increase of 23.29 times; C_dl_ decreases to 25.84% of the untreated surface, which proves that the dielectric coefficient of the corrosion interface interlayer decreases; R_w_ increases significantly to 121 Ω·cm^2^. The results of the electrochemical impedance spectroscopy are consistent with the polarization curves, both indicating that corrosion inhibition of tannic acid and superhydrophobic modification of stearic acid has a significant effect on synergistic protection and enhances the corrosion resistance of the rusted iron samples.

### 3.3. The Mechanism of Superhydrophobic Modification of Rusted Surfaces

#### 3.3.1. Surface Morphological Changes

**Figure 5:**
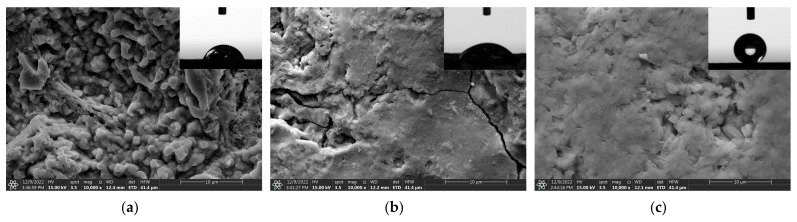
SEM image of an untreated (**a**) corrosion inhibited surface; (**b**) superhydrophobic surface (**c**) of rusted iron samples.

Figure 5 is the scanning electron microscope images showing the microscopic morphology changes of rusted iron samples after different treatments, and the corresponding contact angle photographs. As can be observed from the figure, for untreated surfaces, the rust shows block and rod-like distribution with rust particles of different sizes. The water droplets are nearly hemispherical on the surface with a certain degree of wettability. After the tannic acid immersion treatment, the surface is covered with a protective layer of iron tannate, but there are microcracks on the surface of the film layer, and the coverage is not uniform. The corrosion-inhibited film layer shows stronger hydrophilicity, which is related to the tannic acid containing more hydroxyl groups. After having been modified by stearic acid immersion, the film layer is in the form of a wax-like lamellar with a strong hydrophobic surface, and water droplets form spheres on the surface, effectively isolating from liquid water.

#### 3.3.2. Infrared Spectroscopy

**Figure 6:**
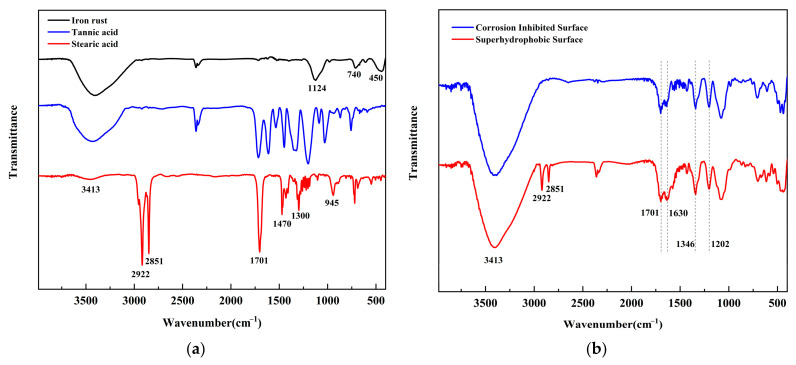
FTIR of (**a**) iron rust, tannic acid and stearic acid; (**b**) rust powder after treatment.

Rust powder in different states is analyzed by infrared spectroscopy in order to examine the chemical structural changes of the rusted surface before and after protective treatment. Figure 6a shows the infrared spectra of rust powder scraped from an untreated iron surface, tannic acid and stearic acid. For the infrared spectrum of rust powder, the peak 3400 cm^−1^ is attributed to the stretching vibration of –OH, the peak 1124 cm^−1^, 740 cm^−1^, 450 cm^−1^ is characteristic of γ-FeOOH, indicating that the rust products on untreated rusted surfaces are mainly γ-FeOOH. For the infrared spectrum of tannic acid, the peak 3400 cm^−1^ is attributed to the stretching vibration of –OH, 1701 cm^−1^, which is attributed to the stretching vibration of C=O; 1600–1400 cm^−1^ is characteristic of the benzene ring; 1346 cm^−1^ and 1202 cm^−1^ could be assigned to the stretching vibration peak of C-O and C-C. For the infrared spectrum of stearic acid, the characteristic absorption peaks of stearic acid include the stretching vibration peaks of sp^3^ C-H at 2851 cm^−1^ and 2922 cm^−1^, and the characteristic peaks at 1470 cm^−1^, 1300 cm^−1^ and 945 cm^−1^.

The IR spectra of the rust powder after protection treatment are shown in Figure 6b. It can be seen that the characteristic peaks of the pure rust powder at 1132 cm^−1^ disappear after tannic acid treatment, and the characteristic peaks of tannic acid at 1202 cm^−1^, 1701 cm^−1^ and 1630 cm^−1^ appear, constituting strong evidence of chemical complexation of tannic acid with trivalent iron. After further stearic acid modification, the telescopic vibrational peaks of stearic acid sp^3^ C-H appeared at 2851 cm^−1^ and 2922 cm^−1^, indicating the effective binding of stearic acid to the rusted surface. In summary, the IR spectroscopy results demonstrate that the water-soluble tannic acid chemically combines with iron hydroxyoxide, and stearic acid effectively binds to iron hydroxyoxide and iron tannate to achieve the superhydrophobic modification effect.

#### 3.3.3. X-ray Photoelectron Spectroscopy

**Figure 7:**
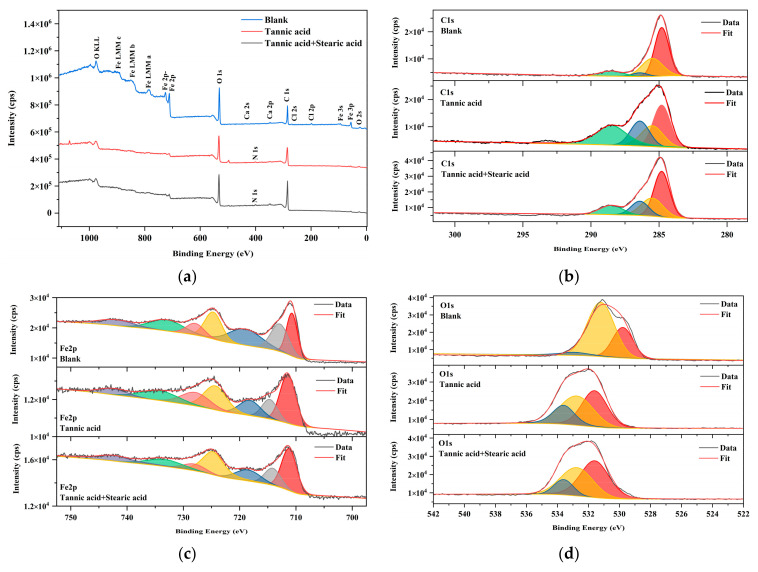
XPS spectra of rusted surfaces after different treatments: (**a**) full spectrum; (**b**) C 1s; (**c**) Fe 2p; (**d**) O 1s.

Iron samples are analyzed by X-ray photoelectron spectroscopy in order to further investigate the mechanism between tannic acid, stearic acid and the rust layer, and to understand the chemical state of the main elements on the rusted surface before and after the protective treatment. Figure 7 shows the XPS spectra of the three samples in the binding energy range of 0 to 1100 eV. Table 3 shows the content of the major elements on three surfaces.

As shown in Figure 7a, the XPS spectra of rusted iron samples contain three elements: the C 1 s at 284 eV, O 1 s at 530 eV, Fe 2p at 711 eV. The content of other elements is very low, and the influence is negligible. Table 3 shows the change in carbon atomic content relative to iron atomic content on the surface, expressed as the atomic C/Fe ratio. For superhydrophobic surfaces, the relative content of carbon elements on the surface increases significantly, the relative content of iron elements decreases significantly, and the C/Fe ratio increases from 0.21 to 2.10, indicating that tannic acid and stearic acid bind to the rusted iron surface and cover it efficiently.

The C 1s peak is decomposed to further analyze the chemical state of carbon on the surface of these samples. In Figure 7b, the detail of the C 1s spectrum is fitted into four peaks with binding energies at 284.8 eV, 285.5 eV, 286.4 eV, and 288.5 eV, respectively. A main peak at 284.8 eV is attributed to a C-C(H) bond, the peak at 285.5 eV and 286.4 eV is attributed to C-O and C=O bonds, and the other peak at 288.5 eV represents O-C=O bonds [37]. It can be seen that the relative content of ester groups is significantly increased after the dehydration and condensation of the carboxyl group of stearic acid and the hydroxyl group on the surface of rusted iron samples to form an ester bond.

As a result of the spin-orbit splitting in 3d transition metals, the high-resolution spectrum of Fe 2p in Figure 7c is divided into two regions, Fe 3p_3/2_ and Fe 2p_1/2_ 710.71 eV corresponds to the Fe 2p_3/2_ characteristic peak of Fe^2+^, such as FeO, and 712.88 eV corresponds to the Fe 2p_3/2_ characteristic peak of Fe^3+^, such as Fe_2_O_3_ and FeOOH [38]. After protective treatment, the intensity of the Fe 2p_3/2_ characteristic peak decreases significantly and shifts towards higher binding energy.

In Figure 7d, the O 1s spectrum is fitted into three peaks. For untreated surfaces, the peak at 529.89 eV is attributed to iron oxides, such as Fe_2_O_3_ and Fe_3_O_4_, and the other two peaks at 531.20 eV and 532.90 eV are attributed to O=C and –OH. For protected surfaces, the three peaks at 531.60 eV, 532.75 eV and 533.60 eV are attributed to O=C, -OH and O-C=O, respectively. It can be seen that the relative content of –OH is significantly increased after the tannic acid containing polyphenolic hydroxyl groups binds to the rusted surface, and the relative content of O-C=O is significantly increased after superhydrophobic modification. Meanwhile, the peak of O1s is shifted towards higher binding energy, which provides additional support for the effective combination of the rust layer, tannic acid and stearic acid.

XPS spectra show that, for superhydrophobic surfaces, the content of the carbon element is significantly increased, the content of the iron element decreased, and the C/Fe ratio increased from 0.21 to 2.10. Meanwhile, the peaks of O 1s and Fe 2p_3/2_ move toward higher binding energy, indicating that tannic acid and stearic acid have achieved effective coverage on the rusted surface. In addition, after the superhydrophobic modification by tannic acid and steric acid, the proportion of C and O was significantly elevated, as seen in Table 3, which proved that the alkyl chain structure of stearic acid successfully covered the outer layer of iron tannate. As mentioned above, a wax-like lamellar substance was formed on the surface under SEM. These results are in agreement with those detected by IR and SEM.

### 3.4. Corrosion Resistance of Superhydrophobic Surfaces

**Figure 8:**
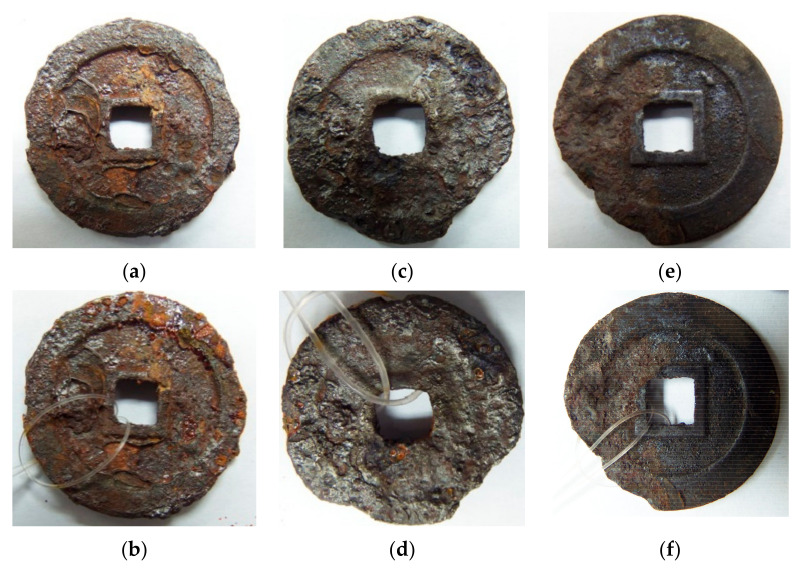
Before and after salt spraying: (**a**,**b**) untreated surface; (**c**,**d**) corrosion inhibited surface; (**e**,**f**) superhydrophobic surface.

In order to test the actual corrosion resistance of rusted surfaces after protective treatment, real artifacts—rusted ancient iron coins—are used as samples for alternating salt spraying experiments, and the results are shown in Figure 8. The most direct qualitative assessment of the corrosion resistance of the protective layer can be made by comparing the amount of new bright orange rust produced on the surface of the iron coin during the hanging process. From the experimental results, it can be seen that before hanging, for the untreated surface, there is a large amount of unevenly distributed orange rust, and the color of the iron coins become significantly darker after corrosion inhibition and superhydrophobic modification. After five cycles of salt spraying, the surface of untreated iron coins produces a large amount of fresh orange rust, which is concentrated at the convexity of the inner and outer edges. The corrosion resistance of the iron coins is significantly improved after the protection treatment. A small amount of fresh rust is generated on the corrosion inhibited surface, while almost no visible signs of fresh rust are produced on the superhydrophobic surface. In high relative humidity with salt, the water film on the surface of untreated iron coins forms an electrolyte solution. The iron undergoes rapid electrochemical corrosion and the iron coins are destroyed. Constructing a superhydrophobic protective layer can effectively prevent the infiltration of liquid water on the surface of iron coins, and the significant decrease of contact area between liquid water and the metal matrix further enhances the corrosion resistance of rusted iron artifacts in high humidity environments.

## 4. Conclusions

The main objective of this article is to provide an easy-to-use, effective material for the corrosion inhibiting and sealing treatment of rusted iron artifacts. Through the joint action of corrosion inhibition of tannic acid and modification of stearic acid, the superhydrophobic layer is successfully constructed on the rusted iron surface, and the water contact angle can reach 152.2°. For the superhydrophobic surface, the number of carbon elements is significantly increased, the content of the iron element decreased, and the C/Fe ratio increased from 0.21 to 2.10. Meanwhile, the peaks of O 1s and Fe 2p_3/2_ move toward higher binding energy, indicating that tannic acid and stearic acid have achieved effective coverage on the rusted surface.

The superhydrophobic surface forms a wax-like lamellar layer as an air barrier to isolate liquid water, resulting in a significant decrease in corrosion current and an increase in Warburg impedance to 217.9 times the original state, with a protection efficiency of 88.3%. In alternating salt spraying conditions, the treatment can also effectively prevent liquid water infiltration on the rusted surface and enhance the corrosion resistance of rusted iron artifacts after corrosion inhibition. Corrosion inhibition of tannic acid and superhydrophobic modification of stearic acid creates a good level of synergistic protection and enhances the corrosion resistance of the rusted iron samples.

## Figures and Tables

**Table 1:** Fitted parameters of polarization curves of rusted iron samples before and after protection.

Samples	J_corr_ /(μA·cm^−2^)	E_corr_ /mV	R_p_ /(Ω·cm^2^)	ν_corr_ /(mm·a^−1^)	β_A_/mV	β_C_/mV
Untreated Surface	111.51	−680.28	233.95	1.3116	334.54	−77.212
Corrosion Inhibited	80.194	−609.59	325.3	0.94326	318.27	−92.552
Superhydrophobic Surface	13.009	−441.83	2005.3	0.15302	200.82	−197.65

**Table 2:** Fitted data of impedance parameters in equivalent circuits.

Samples	R_s_ /(Ω·cm^2^)	R_ct_ /(Ω·cm^2^)	C_dl_ /(mF·cm^−2^)	R_f_ /(Ω·cm^2^)	C_f_ /(μF·cm^−2^)	R_w_ /(Ω·cm^2^)
Untreated Surface	17.04	3.537	15.757	7.154	0.0592	0.558
Corrosion Inhibited	1.253	10.32	18.72	32.16	0.53642	12.72
Superhydrophobic Surface	6.112	82.38	4.0725	77.72	1.2954	121.6

**Table 3:** Fitted data of impedance parameters in equivalent circuits.

Samples	Element	Relative Content	C/Fe
Untreated Surface	C1s	12.63	0.21
	O1s	27.96	
	Fe2p	59.41	
Corrosion Inhibited Surface	C1s	36.59	2.34
	O1s	47.75	
	Fe2p	15.66	
Superhydrophobic Surface	C1s	39.49	2.10
	O1s	41.75	
	Fe2p	18.76	

## Data Availability

The data presented in this study are available in the article.
